# Revisiting: “A pre-post study of a multi-country scale up of resuscitation training of facility birth attendants: does helping babies breathe training save lives?”

**DOI:** 10.1186/s12884-019-2476-3

**Published:** 2019-10-24

**Authors:** Sara Berkelhamer, Nalini Singhal

**Affiliations:** 1 Clinical Associated Professor of Pediatrics, University at Buffalo SUNY, Buffalo, USA; 2 University of Calgary, Calgary, Canada

**Keywords:** Helping Babies Breathe, Resuscitation, Resuscitation training, Neonatal mortality

## Abstract

**Background:**

Helping Babies Breathe (HBB) is a low cost, skills-based neonatal resuscitation education program designed specifically for use in low resource settings. Studies from Tanzania, India and Nepal have demonstrated that HBB training results in decreased rates of fresh still birth and/or neonatal mortality. However, less is known regarding the impact of training on neonatal mortality at a population level. Bellad et al. utilized (BMC Pregnancy Childbirth. 2016;16 (1):222) utilized population based registries to evaluate outcomes before and after training of facility birth attendants. Their study entitled “*A pre-post study of a multi-country scale up of resuscitation training of facility birth attendants: Does Helping Babies Breathe training save lives?*” suggested facility based training was not associated with consistent improvements in neonatal mortality on a population level.

**Discussion:**

Combining outcomes from three diverse settings may have under-estimated the impact of HBB training. We remain concerned that the modest benefits observed in the Kenyan site were lost with compiling of data.

**Summary:**

The statement that HBB “was not associated with consistent improvements in mortality” may lead to the mistaken conclusion that improvements in neonatal mortality were not seen, when in fact, they were in selected cohorts. With numerous studies demonstrating potential for reduced neonatal mortality as a result of HBB training, we encourage interpretation of these findings in the context of local care.

## Main text

We remain concerned about the article published by Bellad, et al. entitled “*A pre-post study of a multi-country scale up of resuscitation training of facility birth attendants: does Helping Babies Breathe training save lives?*“ [1] This multinational study prospectively enrolled over 70,000 births in India (two sites: Belgaum and Nagpur) and Kenya to determine if facility-based implementation of Helping Babies Breathe (HBB) impacted neonatal/perinatal mortality on a population level. While the authors have asked a critical question, their conclusions about the impact of HBB training at a population level across three very diverse settings may cause the reader to under-estimate the potential impact of HBB training on mortality in cohorts most likely to benefit.

In post-hoc analyses, they reported increased survival among infants in Belgaum with birth weights < 2500 g. However, they failed to comment upon probable benefits of HBB training in the Kenyan cohort among infants with birth weights > 1500 g (Table 2 [1]). Modest reduction in both perinatal deaths (38.5 to 28.2/1000, *p* = 0.04) and fresh stillbirth (25.7 to 16.4/1000, *p* = 0.03) in HBB-trained facilities were observed. This may be a critical finding because of the similarity between the Kenyan site and many birth environments in sub-Saharan Africa. The lack of similar benefit in the Indian sites may be attributable to the type of birth attendant; the majority of these births were attended by physicians. It is possible that physicians may have had high baseline knowledge and skill, limiting the potential benefit of additional training.

The study also was designed to “test the possibility that by improving care infants received in facilities, an increasing number of expectant mothers would choose to deliver in facilities” potentially resulting in a “reduction in overall neonatal mortality at the population level.” The authors report that “a large percentage of infants are born outside of health facilities” in low and middle income countries and that “a major emphasis of the World Health Organization has been to encourage women to deliver in a health facility with a skilled birth attendant.” Demonstrating this benefit in two of the three sites, Nagpur and Belgaum, was unlikely because of very high rates of facility births (over 90%) prior to the training. However, there was a modest reduction in home deliveries in Kenya (from 58.1 to 51.3%) following training.

Another aspect of this study was not thoroughly explored. Significant changes in practices and subsequent outcomes following training may be time-dependent. Indeed, prior studies evaluating the impact of training in essential newborn care have shown a decline in perinatal mortality that was independently associated with time since training [2]. The authors acknowledge this concern, explaining that they “set a high bar” by testing “a large effect over a relatively short time period”. The decline in perinatal deaths, fresh stillbirths and deaths by day 1 (Figure 2 [1]) suggests trends over time following the intervention. An analysis with time as a variable would be of interest. A longer follow-up may have ultimately demonstrated improved population outcomes.

The conclusion that HBB “was not associated with consistent improvements in mortality” as stated in the abstract may lead to the mistaken conclusion that improvements in neonatal mortality were not seen, when in fact, they were in selected cohorts. With numerous studies, including this one, demonstrating potential for some reduction of neonatal mortality as a result of HBB training, we encourage interpretation of these findings in the context of local care [3, 4].

## Response

Linda L Wright^3^, Akash Bang^4^, Elizabeth M McClure^5^ and Waldemar A. Carlo^6^.

^3^*Eunice Kennedy Shriver* National Institute of Child Health and Human Development, Bethesda, MD, USA (ret).

^4^Mahatma Gandhi Institute of Medical Sciences, Sewagram, India.

^5^RTI International, Durham, NC.

^6^University of Alabama at Birmingham, Birmingham, AL, USA.

Berkelhamer and Singhal raise concerns about the validity of our conclusion that HBB was not associated with consistent improvements in mortality in our study populations [1]. They raise two specific concerns with the conclusions from the manuscript. First, they suggest that the statement that “HBB was not associated with consistent improvements in mortality,” as stated in the abstract, “may lead to the mistaken conclusion that improvements in neonatal mortality were not seen, when in fact, they were in selected cohorts.” This conclusion appears to be based on their evaluation of the results from the Kenya site, where they conclude, “Reduction in both perinatal deaths (38.5 to 28.2/1000; *p*=0.04) and fresh stillbirths (25.7 to 16.4/1000, *p*=0.03) in HBB-trained facilities were observed.” Second, they express concerns, consistent with the cautions noted in the manuscript, that practice changes may take time and that the study was of insufficient length to show the benefits of these practice changes, specifically noting, “The decline in perinatal deaths, fresh stillbirths and deaths by day 1 suggests trends over time following the intervention. Analyses with time as a variable would be of interest.”

We appreciate the concerns Berkelhamer and Singhal raise about the limitations of our study design to identify effects that may become apparent at 2 to 3 years or beyond. However, we stand by our original conclusion that “HBB was not associated with consistent improvements in mortality in our study populations.” Furthermore, we are concerned that the Berkelhamer and Singhal’s conclusions are overly optimistic and inconsistent with established statistical practice and guidelines for interpreting clinical trial results. The results on reduction in perinatal deaths and fresh stillbirth cited from Kenya are post hoc site-specific subpopulation results that must be interpreted extremely cautiously in the light of the complete absence of a site-by-treatment interaction (*p* > 0.33 as noted in our paper). Drawing conclusions from the site-specific *p*-values in the absence of a site-by-treatment interaction is inconsistent with the CONSORT guidelines for interpretation of clinical trials which specifically note, “When evaluating a subgroup the question is not whether the subgroup shows a statistically significant result but whether the subgroup treatment effects are significantly different from each other.” [5] Furthermore, given the large number of secondary subgroup comparisons presented in Tables 2 and 3 [1], a substantially smaller *p*-value (*p* < 0.001) would be required to provide reasonable evidence of a cohort effect, in contrast to the modest p-value found for the Kenya results. While we acknowledge that our study was not designed to assess delayed impacts of the intervention, we disagree that the results in Figure 2 [1] provide any evidence of a downward trend given the substantial overlap of the confidence intervals over the full pre- and post-period. Because training effects declined over time [6], a study of longer duration would have been unlikely to show a benefit.

## Data Availability

Not applicable

## Abbreviation

HBB: Helping Babies Breathe
